# Uptake of hepatitis B-HIV co-infection screening and management in a resource limited setting

**DOI:** 10.1186/s41124-017-0030-3

**Published:** 2018-01-06

**Authors:** Musomba Rachel, Castelnuovo Barbara, Claire Murphy, Charlene Komujuni, Patience Nyakato, Ponsiano Ocama, Mohammed Lamorde, Philippa Easterbrook, Rosalind Parkes Ratanshi

**Affiliations:** 1 Infectious Diseases Institute, Makerere University, Mulago Hospital, P.O. Box 22418, Kampala, Uganda; 2 Centre for Communicable Diseases, Queen Elizabeth University Hospital, Glasgow, Scotland; 3 World Health Organizations, Geneva, Switzerland; 4 Cambridge Institute of Public Health, Cambridge, UK

**Keywords:** Hepatitis B, HIV/AIDs, Resource limited setting

## Abstract

**Background:**

WHO hepatitis B guidelines recommend testing all new HIV patients, treating them accordingly or providing immunization. At the Infectious Diseases Institute (IDI) following an audit done in 2012, only 46% patients had been screened for hepatitis B with variable management plans therefore new internal guidelines were implemented. This study describes the uptake of hepatitis B screening and management of patients with hepatitis B and HIV con-infection after the implementation.

**Methods:**

Data included for all HIV positive patients in care at IDI by October 2015. Data are expressed as median with interquartile range (IQR) and percentages were compared using the chi square test. Statistical analysis was performed using STATA version 13. The IDI laboratory upper limit of normal for alanine aminotransferase (ALT) and aspartate aminotransferase (ASTs) was 40 IU/ml.

**Results:**

Number of hepatitis B screening tests increased from 800 by 2012 to 1400 in 2015. By 2015 8042/8604(93.5%) patients had been screened for hepatitis B. Overall hepatitis B positive were 359 (4.6%). 166 (81.4%) hepatitis B positives were switched to a tenofovir (TDF) containing regimen.

**Conclusion:**

Our study confirms the importance of screening for hepatitis B and of using ART regimens containing tenofovir in hepatitis B co-infected patients. Whilst our program has made improvements in care still 18.6% of patients with hepatitis B were not on tenofovir regimens, 98.1% had no hepatitis B viral loads done. Clinicians should recognize the potential for hepatitis B in HIV positive patients and the importance of early diagnosis and treatment to ensure optimal management of cases and follow up.

## Background

Chronic viral hepatitis is an increasing cause of morbidity and mortality among HIV confected persons, including those on ART, as persons with HIV continue to live longer [1–3]. The consequences of coinfection include higher rates of chronicity, less spontaneous clearance, accelerated fibrosis progression with increased risk of cirrhosis and hepatocellular carcinoma, higher liver-related mortality, and decreased treatment response [4–6]. Other challenges with coinfection include cross-resistance between HIV and hepatitis B drugs, [7–9] and increased rates of hepatoxicity [10, 11].

Chronic hepatitis B virus infection affects 5–20% of the 36 million people living with HIV worldwide, and the burden of HIV- hepatitis B co-infection is greatest in low and middle income countries, particularly in sub-Saharan Africa. Globally it is estimated that 2.6 million are HIV- hepatitis B co-infected [12]. The World Health Organization (WHO) in an effort to prevent and control hepatitis B Infection has developed a global action framework with four strategic axes: 1) raising awareness, promoting partnerships, and mobilizing resources 2) evidence-based policy and data for action 3) prevention of transmission 4) screening, care and treatment. These strategies offer a global vision for the prevention and control of viral hepatitis [13, 14].

Uganda is highly endemic for hepatitis B infection, with a seroprevalence for hepatitis B positivity of around 10%, based on a national hepatitis serosurvey in 2005 [15, 16]. As with most of Sub-Saharan Africa, the majority of infection is acquired at birth or during early childhood (Franco E, 2012). In 2002, Uganda adopted a policy of providing hepatitis B vaccination to all infants [17] . Uganda has also adopted the 2013 WHO guidelines [18] that recommend hepatitis B testing in all HIV infected patients,and particularly those with elevated alanine aminotransferase (ALT), with linkage to care and treatment, and vaccination if Hepatitis B negative. Additionally knowledge of Hepatitis B serostatus allows optimal selection of antiretroviral drug (ARV) regimen to one that includes tenofovir plus lamivudine or emtricitabine (TDF/3TC or FTC) to ensure additional activity against hepatitis B [18, 19]. During the study period, the Ugandan Ministry of Health recommendations were for immediate initiation of ART for all patients with a CD4 count less than 350 cells, and less than 500 for those with hepatitis B HIV co-infection [20].

Whilst access to HIV care is becoming widely available in Sub-Saharan Africa, in diagnosis and treatment of viral hepatitis co-infection there remains a big disparity between resource rich and poor settings. Limitations such as the availability of further investigations and medication can be challenging in these settings to provide individualized care for more complex patients, such as those with co-infection [21–24].

An audit conducted in September 2012 among newly registered patients at the Infectious Diseases Institute – a large urban HIV clinic in Kampala, Uganda showed a low uptake of hepatitis B screening and variable management of those diagnosed Hepatitis B positive [25]. In response; a new specialty clinic was set up with the aim of identifying and appropriately care for patients co-infected with HIV and hepatitis B.

This study describes the impact on uptake of hepatitis B testing, assesses whether new guidelines and the introduction of the hepatitis B specialty clinic increased hepatitis B screening uptake and improved clinical management.

## Methods

### Study site and development of hepatitis B clinic services and policies

The Infectious Diseases Institute (IDI) is an HIV center of excellence [26] located in Mulago National referral hospital in Kampala, Uganda. IDI was one of the first HIV care and treatment organizations in Uganda; the IDI clinic began providing HIV care in 2002, while free antiretroviral treatment has been provided since April 2004. In 2015 there were more than 8000 HIV infected patients in active care.

#### Timescale of key activities relating to delivery of hepatitis B testing and treatment at IDI

1. In 2010, routine hepatitis B testing was first introduced, followed in 2012 by establishment of a dedicated hepatitis B clinic and clinic guidelines. The new guidelines were written by a team of three doctors, the quality manager and approved by the senior Physician who is also the head of the Prevention, Care and Treatment (PCT) department. One staff training session was conducted and the guidelines were implemented starting 1st September 2012. The hepatitis B clinic is held every Friday, staffed by doctors, nurses, counselors, laboratory technician, and pharmacist.
2. IDI has an electronic patient management system (ICEA) [27] and since the new guidelines were introduced in 2012, an electronic alert on the system that reminds clinicians to request hepatitis B tests for patients who have not been screened were further developed, the reminder prompted clinicians to request for the test. Samples were tested for hepatitis B using the hepatitis B surface antigen. Those with a positive test were termed hepatitis B positive. Routine hepatitis core antigen testing was not available.
3. Since September 2012, in accordance with the new IDI clinic guidelines, all newly registered HIV patients are tested for hepatitis B surface antigen, while those already registered in care are tested during their routine clinic appointments. ART naive hepatitis B positive patients are prepared to start ART with two pre-ART counselling sessions. In hepatitis B positive patients already on a zidovudine (AZT) based ART regimens, zidovudine (AZT) is substituted for tenofovir (TDF) if the viral load is undetectable, or switched to a second line regimen containing tenofovir (TDF), if viral failure is confirmed. Free hepatitis B testing and treatment is provided, but additional investigations including hepatitis B DNA viral load and fibro scan to help stage liver disease and monitor response to treatment; ultrasonography and alpha-fetoprotein measurement to screen for hepatocellular carcinoma; and endoscopy to screen and manage varices due to hepatitis B infection related complications need to be paid for by the patient. However, these are expensive and beyond the financial means of many of our patients.

### Study population and data collection

This analysis includes all adult (≥18 years) HIV patients in current care at IDI from January 2002 to October 2015, with censorship at lost to follow up (LFU), transfer, or death. For each patient we recorded demographic, clinical, ART status, hepatitis B status, liver function test results, clinical management decisions regarding use of tenofovir containing ART regimen, and clinical outcomes (death, LFU, transfer etc). We also describe the number of patients tested each year, and compared the clinical characteristics and outcomes between those who tested hepatitis B positive and negative.

Two audits were undertaken for this study; the first was done in September 2012 and looked at newly enrolled patients, the second was undertaken in October 2015 following the establishment of the hepatitis B-HIV clinic.

### Statistical analysis

We describe the number of patients tested each year and compared baseline characteristics (age, gender, WHO stage) in hepatitis B positive and negative patients and described the clinical outcomes in hepatitis B positive patients. Data are expressed as median with interquartile range (IQR) or number and percentages as appropriate for (CD4 count at hepatitis B test, ART status, current ART regimen, time in days from ART start to hepatitis B test). Proportion of hepatitis B co-infected patients already on a TDF containing ART regimen at the time of test are described. To show trends of for patients tested before and after guideline implementation, we used proportion of patients tested for hepatitis B compared to the number of patients who registered in the period. *P* values were found using Cochran-Armitage test for trend. We used an IDI laboratory upper limit of normal for alanine aminotransferase (ALT) and aspartate aminotransferase (ASTs) of 40 IU/ml. We used the t test for comparison of parametric continuous variables, Wilkinson rank sum test for non-parametric variables and chi square and fisher’s exact tests for categorical variables. Statistical analysis was performed using STATA version 13.

## Results

In the first audit, 1753 patients were reviewed. Of these only eight hundred seven patients (46%) received hepatitis B testing. Twenty-nine (3.6%) of those tested were hepatitis B positive, of which 8/29 (27.6%) were not started on ART; of note five of those had CD4 count < 500 cells/μL. Of 21 who started ART, eighteen (62.1%) were prescribed TDF-3TC containing regimens, three (10.3%) on ART regimens not known to have activity against hepatitis B. 19/29 (65.5%) patients had baseline liver function tests (LFTs) checked. No patients had follow up LFTs.

**Fig. 1:**
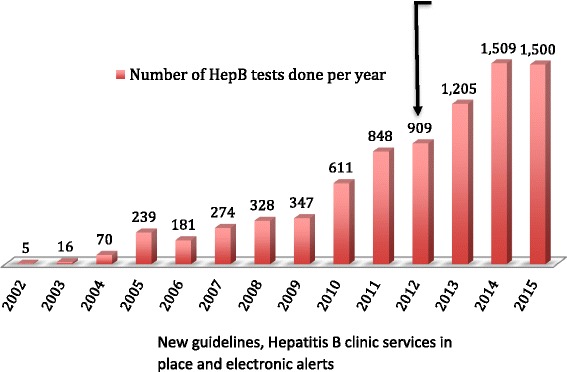
Number of hepatitis B tests done performed per each year in IDI from January 2002 to October 2015

During the repeat audit in October 2015, there were 8042 (93.5%) active HIV infected patients screened for hepatitis B at IDI. Of these,2984(37.1%) were male, with median age 31 years (IQR: 26–35), majority in WHO stage 3 and 4; 4608(57.3%). 7416(92.2%) were initiated on ART and were on first line ART regimen 6930 (86.2%). There has been a marked increase in the uptake of testing per year from 611 tests in 2010, to 909 in 2012 and now 1500 in 2015. Overall, 8042 (93.5%) of 8604 current HIV infected patients have been screened for hepatitis B with the largest number of patients being screened between 2014 and 2015. Figure 1 shows the number of hepatitis B tests done each year over the last twelve years.

The proportion of patients screened for hepatitis B compared to the number of patients newly registered in the clinic was higher after the implementation of the new guidelines (2141(79.3%) of 2700 versus 5901 (28.4%) of 20,774 before the guidelines *p* < 0.001).

### Characteristics of HIV-infected persons with and without hepatitis B infection as of October 2015

Table 1 shows the descriptive characteristics of 359 (4.6%) hepatitis B positive patients compared to the 7083 hepatitis B negative.

**Table 1:** Demographic and clinical characteristics among hepatitis B positive and negative patients

Characteristics	Hepatitis B positive *N* = 359	Hepatitis B negative *N* = 7683	*P* value
Male gender N (%)	189 (52.6%)	2795 (36.4%)	<0.0001
Median Age (IQR) in years	38 (32, 45)	39 (32, 46)	0.119
WHO stage N (%) 3&4	220 (61.3%)	4388 (57.1%)	0.156
Median CD4 count at hepatitis B test (IQR) cells/μL	423 (269, 600)	483 (325, 657)	<0.0001
Ever used ART	337 (93.9%)	7079(92.1%)	0.231
Current ART regimen N (%)			
First line	316 (88.0%)	6569 (85.5%)	0.101
Second line ART	20 (5.6%)	507 (6.6%)	
Third line ART	1 (0.3%)	1 (0.01%)	
Median time (days, IQR) from ART start to hepatitis B test	74 (27, 399)	105 (28, 596)	< 0.0001
Active	289 (83.2%)	6801 (88.5%)	<0.0001
Dead	23 (6.5%)	179 (2.3%)	
Lost	5 (1.4%)	118 (1.5%)	
Transferred out	33 (9.6%)	585 (7.7%)	

Those who were hepatitis B positive, were more likely to be male (52.6%) versus (36.4%) (*P* < 0.0001) and to have a lower median CD4 count at hepatitis B test (423 cells/μL) (IQR 269–600) versus 483 cells/μL (IQR 325–657). In addition, hepatitis B positive had been on ART for shorter period (days 74 (IQR 27, 399) vs (105 (IQR 28, 596) days) at the time of the hepatitis B test.

There was no difference in median age at 38 (IQR) or in WHO stage 3 and 4 (61.3%). Three hundred thirty-seven (93.9%) of the hepatitis B positives ever used ART with a median time on ART seventy-four days from hepatitis B test (IQR 27–399). Two hundred fifty-four (75.4%) patients started ART before the hepatitis B test date, eighty-one (24.1%) after the test and two (0.5%) on the day of the test. The proportion of deaths (6.5%) was higher among the hepatitis B positives as compared to the hepatitis B negative population (2.3%) (*p* value < 0.0001).

The median time from testing positive for hepatitis B test to death was 143 days (IQR 15–242). Hepatitis B positive patients were on ART for shorter period prior to testing (days 74 (IQR 27, 399) as compared to the hepatitis B negative patients (105 (IQR 28, 596) days) at the time of the hepatitis B test. Hepatitis B positive had lower CD4 count: 423 cells/μL, (IQR: 269–600) compared to hepatitis B negative patients 483 cells/μL (IQR:325, 657), *p* value < 0.0001).

### Clinical management

**Fig. 2:**
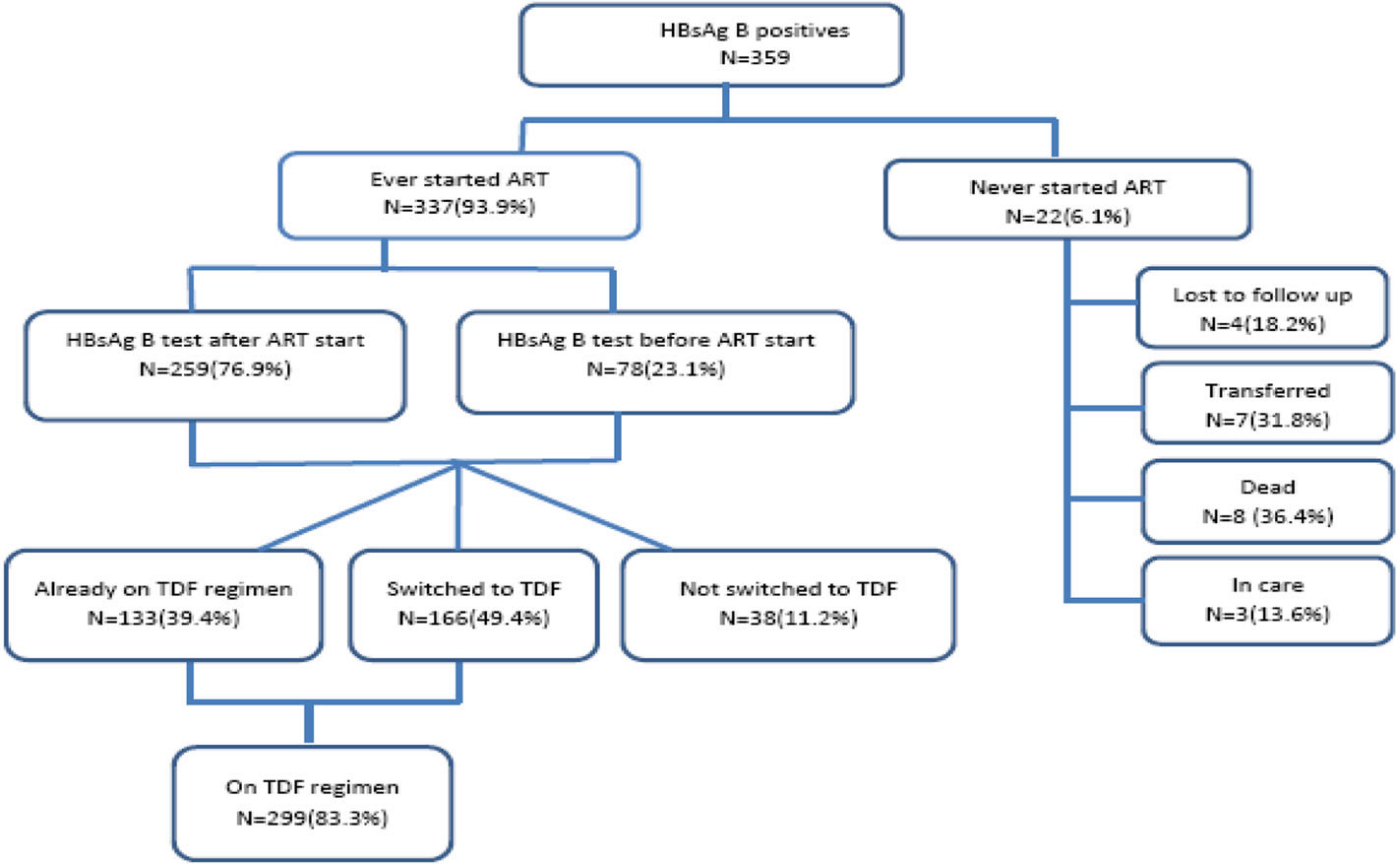
Summarizes the management sequence for the 359 HIV infected persons identified as hepatitis B positive, as of October 2015

Figure 2. Management sequence for the 359 HIV-hepatitis B positive persons, as of October 2015

### ART management

337 (93.9%) had been started on ART, while 22 (6.1%) had not been started not on ART at the time of the analysis. Of the 22 not on ART - most died (8, 36.4%), four (18.2%) were lost to follow up, seven (31.8%) had been transferred out while three (13.6%) were active in care. One patient of those active in care had documented evidence that they refused to initiate ART while two had results returned before their scheduled return clinic appointments date. Overall 259(76.9%) had been initiated on ART prior to their hepatitis B test, and 78 (23.105%) after.

One hundred thirty-three of 337 (39.4%) patients were already on a TDF containing ART regimen at the time of test, 166 of 337 (49.3%) were subsequently switched to TDF within median of 3.7 months (IQR 1.8–6.3) from hepatitis B test, and 38 (11.2%) were never switched. The most commonly documented reason for not switching was that the viral load was ˂1000 copies/ml for 24 (92.3%) hepatitis B patients.

By October 2015, two hundred ninety nine (83.3%) of 359 patients were on a TDF based regimen.

### Investigations and status of liver disease

Overall 285 (79.6%) hepatitis B positives had an HIV viral load done with 253 (70.7%) of patients with viral load VL˂1000 copies/ml. Patients who never had an HIV viral load done 74(20.4%), 24 (32.9%) patients were active in care, 17 (23.4%) were dead, 4 (5.5%) were lost from care while twenty eight (38.4%) had been transferred out to other health care providers.

Liver enzymes tests (ALTs) were done for 249 (69.4%) patients with 205 (82.3%) of patients having normal alanine aminotransferase levels. Patients with Aspartate transaminase (ASTs) test done were 86 (24%) most of which (61.6%) had results in normal ranges.

Patients with hepatitis B DNA viral loads done were 7(1.9%) while 250 (69.6%) patients had an ultra sound scan done.

### Clinical outcomes

Overall twenty three (6.5%) of the hepatitis B positives died, of these 9(39%) were those who had never been initiated on ART.

## Discussion

Our findings show the striking impact of the introduction and implementation of a series of clinic activities to support the provision of effective hepatitis B care in an HIV clinic. There was a progressive increase in the uptake of hepatitis B testing particularly over the preceding four years 79.3%, with now almost universal testing of all HIV infected patients at IDI. It also shows the direct impact of knowledge of hepatitis B status on ART regimen choice with 299(83.3%) of 359 patients who were identified as positive on a TDF regimen as recommended in WHO guidelines [15] and consistent with other studies in the region [28].

This scale up and response was largely achieved as a result of results through actions taken in response to the findings of the first audit in 2012 showing a low uptake of testing [25]. These actions were the creation of a hepatitis B dedicated clinic at the facility; the development of specific guidelines; provision of, intensive training of health care worker staff on the current guidelines; and creation of a hepatitis B dedicated clinic at the facility. Further systems like the development of electronic alerts on the patient management systems (ICEA) [27] to remind clinicians to request for testing on all patients not yet tested.

Our study showed that hepatitis B occurred in 359(4.6%) patients; slightly lower than the general hepatitis B seroprevalence for Kampala regions (5.3%) reported in the 2005 national serosurvey [16, 29, 30]. Additional reasons for the lower prevalence maybe the impact of effective ART with TDF and 3TC/FTC containing regimens which may have resulted in hepatitis B clearance. This concurs with other data from the multicenter AIDS cohort study showing that hepatitis B was significantly lower in the ART era than in the pre-ART (IRR, 0.2 [CI, 0.1 to 0.4]), and that effective ART is associated with lower incidence of hepatitis B infection [31, 32].

Analysis of our study population showed that there were more deaths 23 (6.5%) among the hepatitis B positives compared to the negatives patients 179 (2.3%, *p* value <0.0001). (Table 1) These results are similar to findings from other studies. [2, 3, 33–36].

Further investigations and management of hepatitis B patients is still a challenge. IDI has access to hepatitis B, hepatitis C and E testing only. Further investigations that would be standard in resource rich settings are not available. Additional tests including liver function tests and ultra sound scans for hepatitis B patients were not done for all the patients. Only 1.9% of patients were able to have a hepatitis B DNA viral load. In Uganda generally extensive medical investigations are expensive and beyond the financial means of IDI and many of the patients. In our settings, diagnosing chronic liver disease and hepatitis infection is currently a challenge without external funding and availability of appropriate diagnostic investigations. There are also limited drug combinations for those with drug resistance and for more complex patients, such as those with co-infection.

## Conclusion

Clinicians should recognize the potential for hepatitis B in HIV positive patients and the importance of early diagnosis and treatment to ensure optimal management of cases and follow up. Effective prevention and treatment of hepatitis B virus infection is an important public health priority with effective ART being associated with lower incidence of hepatitis B infection.

The Ugandan government is making progress in prioritizing hepatitis B screening. We advocate for further budgetary allocation for the implementation of hepatitis B treatment, management and long term screening for liver cancer. In the future additional funding is still required to increase management for complex patients with HIV-hepatitis B co-infection and its complications.

## Abbreviations

ART: Antiretroviral therapy
ASTs: Aspartate aminotransferase
DNA: Deoxyribonucleic acid
IDI: Infectious Diseases Institute
LFTs: Liver function tests
